# Frovatriptan versus almotriptan for acute treatment of menstrual migraine: analysis of a double-blind, randomized, cross-over, multicenter, Italian, comparative study

**DOI:** 10.1007/s10194-012-0455-4

**Published:** 2012-05-17

**Authors:** Marco Bartolini, Maria Adelaide Giamberardino, Carlo Lisotto, Paolo Martelletti, Davide Moscato, Biagio Panascia, Lidia Savi, Luigi Alberto Pini, Grazia Sances, Patrizia Santoro, Giorgio Zanchin, Stefano Omboni, Michel D. Ferrari, Brigida Fierro, Filippo Brighina

**Affiliations:** 1 Clinica Neurologica, Ospedali Riuniti, Università Politecnica delle Marche, Ancona, Italy; 2 Dipartimento di Medicina e Scienze dell’Invecchiamento, Università “G. D’Annunzio”, Chieti, Italy; 3 Ospedale Civile San Vito al Tagliamento, San Vito al Tagliamento, Italy; 4 Department of Medical and Molecular Sciences, Sapienza University of Rome and Regional Referral Headache Centre, Sant’Andrea Hospital, Rome, Italy; 5 Headache Centre, San Carlo IDI Hospital, Rome, Italy; 6 Centro Cefalee, A.O. Universitaria Vittorio Emanuele, Catania, Italy; 7 Department of Neurology, University of Torino, Turin, Italy; 8 Centro Cefalee, Università degli Studi di Modena e Reggio Emilia, Reggio Emilia, Italy; 9 Centro Cefalee, IRCCS Neurologico C. Mondino, Pavia, Italy; 10 Clinica Neurologica, Ospedale S. Gerardo, Monza, Italy; 11 Department of Neurology, University of Padova, Padua, Italy; 12 Italian Institute of Telemedicine, Varese, Italy; 13 Department of Neurology, Leiden Centre for Translational Neuroscience, Leiden University Medical Centre, Leiden, The Netherlands; 14 Department of Neurology and Psychiatry, Policlinico of Palermo, University of Palermo, Palermo, Italy

**Keywords:** Migraine, Menstrually related migraine, Frovatriptan, Almotriptan

## Abstract

The objective of the study was to compare the efficacy and safety of frovatriptan and almotriptan in women with menstrually related migraine (IHS Classification of Headache disorders) enrolled in a multicenter, randomized, double-blind, cross-over study. Patients received frovatriptan 2.5 mg or almotriptan 12.5 mg in a randomized sequence: after treating 3 episodes of migraine in no more than 3 months with the first treatment, the patient was switched to the other treatment. 67 of the 96 female patients of the intention-to-treat population of the main study had regular menstrual cycles and were thus included in this subgroup analysis. 77 migraine attacks classified as related to menses were treated with frovatriptan and 78 with almotriptan. Rate of pain relief at 2 and 4 h was 36 and 53 % for frovatriptan and 41 and 50 % for almotriptan (*p* = NS between treatments). Rate of pain free at 2 and 4 h was 19 and 47 % with frovatriptan and 29 and 54 % for almotriptan (*p* = NS). At 24 h, 62 % of frovatriptan-treated and 67 % of almotriptan-treated patients had pain relief, while 60 versus 67 % were pain free (*p* = NS). Recurrence at 24 h was significantly (*p* < 0.05) lower with frovatriptan (8 vs. 21 % almotriptan). This was the case also at 48 h (9 vs. 24 %, *p* < 0.05). Frovatriptan was as effective as almotriptan in the immediate treatment of menstrually related migraine attacks. However, it showed a more favorable sustained effect, as shown by a lower rate of migraine recurrence.

## Introduction

Female migraineurs frequently experience headaches in association with their menstrual cycles [1, 2]. These migraine attacks represent a challenge for both the patient and the headache specialist as they have been shown to be particularly difficult to treat and more disabling than migraines outside of the menstrual period [3].

Triptans are a recommended first-line treatment for moderate to severe migraine, including menstrual migraine [2, 4]. Sumatriptan, the first triptan to be marketed, has been shown to be well tolerated and effective in providing pain relief in menstrual migraine when administered in the mild pain phase, and also when used in combination with analgesics [5–11]. Second generation triptans such as zolmitriptan [12–14], naratriptan [15], rizatriptan [16–20] and more recently almotriptan [13, 21–24] and frovatriptan [25] have also been successfully tested. Most of these drugs have been found to be effective for treatment or prevention of the acute attack of menstrual migraine [26, 27].

Efficacy of frovatriptan in the short-term prevention or acute treatment of menstrual migraine has been demonstrated in several randomized, double-blind, placebo-controlled or open-label studies [28–34]. In two recently published retrospective analyses of randomized, double-blind, cross-over, head-to-head trials, frovatriptan provided a more sustained pain relief than zolmitriptan and rizatriptan, with similar pain-free and pain-relief rates at 2 h [35, 36].

The objective of the present study was to add new evidence to the existing body of data through a subgroup analysis of a double-blind, randomized, cross-over study, comparing frovatriptan with almotriptan in a broader group of migraine patients [37].

## Methods

### Study population

The analysis of the subgroup of women suffering from menstrual-related migraine population was pre-defined in the statistical analysis plan and original protocol of the main study [37]. This condition was defined, according to the Appendix of the International Headache Society (IHS) 2004 [38] research criteria, as migraine without aura attacks in a menstruating woman, occurring on day 1 ± 2 (namely days −2 to +3) of menstruation in at least two out of three menstrual cycles and additionally at other times of the cycle. All subjects had to report at least one, but no more than 6 migraine attacks per month in the 6 months prior to entering the study.

Exclusion criteria are reported in detail in the main study publication [37]. Briefly, women were excluded from the study in case of uncontrolled hypertension, ischemic heart disease, cardiac arrhythmias, previous stroke or transient ischemic attack, severe liver or renal impairment or any other severe or disabling medical condition. History of alcohol or drug abuse, use of anti-migraine drugs, including either test medication for treating one of the last three episodes of migraine, known hypersensitivity to study drugs, previously demonstrated inadequate response to at least two triptans or occurrence of other headaches that have been lasting for more than 6 days, were also considered as exclusion criteria. Pregnancy and breast-feeding precluded participation in the study, while women with childbearing potential but not practicing an effective method of birth control were to be submitted to a pregnancy test, if clinically indicated.

The study was approved by the local institutional review board of the study centers. Written informed consent was provided by all patients before study participation.

### Study design

This was a multicenter, randomized, double-blind, cross-over study, including 14 Italian centers (see "Appendix") [37]. Each patient received frovatriptan 2.5 mg or almotriptan 12.5 mg in a randomized sequence: after treating a maximum of 3 episodes of migraine in no more than 3 months with the first treatment, the patient was switched to the other treatment and was asked to treat a maximum of 3 episodes of migraine in no more than 3 months with the second treatment.

Subjects having no migraine episodes during one of the two observation periods were excluded from the study.

After signing written informed consent, subjects provided a medical, treatment and migraine history. A physical and neurological examination and pregnancy test (if appropriate) were performed. Blood pressure and heart rate were measured for all subjects. The degree of migraine-associated disability (MIDAS) questionnaire was also completed. At the end of the visit a headache diary documenting characteristics of headache pain (including its relation with menses) and associated symptoms was dispensed with study medication to eligible patients. Subjects were instructed to treat a maximum of 3 migraine episodes within a period of no more than 3 months and then to come for the second visit. At this second visit, use of concomitant medications and occurrence of possible adverse events (obtained from diary card) were checked, blood pressure and heart rate were recorded, and a pregnancy test performed, if deemed necessary. At the end of the second visit a headache diary documenting characteristics of headache pain was dispensed with study medication. The same procedures were carried out at the end of the second study treatment period or at the early withdrawal visit.

Patients were instructed to take one dose of study medication as early as possible after the onset of migraine attack. If insufficient relief had been obtained after 2 h, patients were allowed to take a second dose of study medication, with a maximum daily intake of two doses. In case of insufficient relief one hour after the intake of the second dose of the study medication, patients were allowed to take a rescue medication. Rescue medication could not include triptans, or contain ergotamine or its derivatives.

The study involved three visits and each patient’s participation time in the study was not to exceed 6 months from randomization.

Randomization was done by blocks of 4. Blindness was ensured by the overencapsulation technique, i.e., by inserting study drug tablets in capsules.

### Data analysis

The population of this retrospective analysis consisted of all normally menstruating women randomized to any of the two treatment sequences, enrolled to receive either of the study treatments and having treated at least one episode of menstrual migraine with both medications (intention-to-treat population).

The following endpoints were evaluated [37]: (a) the proportion of pain-relief episodes at 2, 4 and 24 h (a decrease in migraine intensity from severe or moderate to mild or none at 2, 4 and 24 h); (b) the proportion of pain-free episodes at 2, 4 and 24 h (absence of migraine episodes at 2, 4 and 24 h after intake of one dose of study drug); (c) recurrence within 24 h (episodes pain free at 2 h and headache of any severity returns within 24 h); (d) recurrence within 48 h. Changes in headache intensity (4-point scale from none to severe) from baseline after 2, 4, 24 and 48 h from study drug intake, were also evaluated.

For continuous variables average values and standard deviation (SD) were calculated. Categorical variables were summarized by computing the absolute value and the frequency (as percentage). Endpoints were compared between groups by generalized estimating equation analysis. Kaplan–Meier curves for cumulative hazard of recurrence over the 48 h were also drawn. All statistical tests were performed using a two-sided test with a significance level of 0.05 (*p* value).

## Results

The main study population consisted of 114 subjects [37], of which 96 were women. 67 of them treated at least one episode of menstrual migraine with both medications and were thus included in the present analysis.

No statistically significant differences were observed for the main demographic and clinical characteristics of the patients of the whole study population and of the subgroup of women with menstrually related migraine (Table 1). The only exception was a significantly (*p* < 0.05) wider use of triptans in the previous 3 months in the women with menstrually related migraine.

**Table 1:** Demographic and clinical baseline data of the 114 patients of the main study [37] and of the subgroup of 67 women with menstrually related migraine

	Main study (*n* = 114)	Menstruating women (*n* = 67)	*p*
Age (years, means ± SD)	40 ± 10	37 ± 8	NS
Height (cm, means ± SD)	165 ± 6	164 ± 6	NS
Weight (kg, means ± SD)	65 ± 12	62 ± 10	NS
Age at onset of migraine (years, means ± SD)	18 ± 8	17 ± 7	NS
Migraine attack duration >2 days (*n*, %)	29 (25)	16 (24)	NS
MIDAS score (means ± SD)	23 ± 16	23 ± 16	NS
No use of triptans in the previous 3 months (*n*, %)	93 (82)	46 (69)	<0.05
Moderate or severe attacks (*n*, %)^a^	532 (80)	133 (86)	NS
Patients with at least one moderate or severe attack (*n*, %)	111 (97)	64 (96)	NS

Data are shown as mean (±SD), or absolute (*n*) and relative frequency (%)

^a^Numbers refer to number and frequency of attacks as respect to overall number of attacks

A total of 77 attacks (24 % of all attacks) classified as menstrually related migraine were treated with frovatriptan and 78 (24 %) with almotriptan.

As indicated in Table 2, the proportion of pain-relief episodes was similar (*p* = NS) between frovatriptan and almotriptan at 2 h (36 vs. 41 %), 4 h (53 vs. 50 %) and 24 h (62 vs. 67 %). Also the rate of pain-free episodes at 2, 4 and 24 h did not differ between the two treatment groups (19, 47 and 60 % frovatriptan vs. 29, 54 and 67 % almotriptan; *p* = NS for both) (Table 2).

**Table 2:** Main study endpoints in the two study treatment groups

	Frovatriptan	Almotriptan	*p*
Pain-relief episodes at 2 h	28 (36)	32 (41)	NS
Pain-free episodes at 2 h	15 (19)	23 (29)	NS
Pain-relief episodes at 4 h	41 (53)	38 (50)	NS
Pain-free episodes at 4 h	36 (47)	42 (54)	NS
Pain-relief episodes at 24 h	48 (62)	53 (67)	NS
Pain-free episodes at 24 h	46 (60)	52 (67)	NS
Recurrent episodes at 24 h	6 (8)	16 (21)	<0.05
Recurrent episodes at 48 h	7 (9)	19 (24)	<0.05

Data are reported as absolute (*n*) and relative (%) frequency.* p* refers to the statistical significance of the difference between the two study drugs

**Fig. 1:**
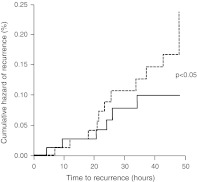
Cumulative hazard of recurrence over the 24 h during treatment with frovatriptan (*continuous line*) or almotriptan (*dashed line*) in the 67 women with menstrually related migraine included in this analysis. The *p* value refers to the statistical significance of the between-treatment difference

The rate of migraine recurrence after 24 h was lower (*p* < 0.05) with frovatriptan than with almotriptan (8 vs. 21 %). This was the case also for recurrences at 48 h (9 % frovatriptan vs. 24 % almotriptan, *p* < 0.05). Also, the cumulative hazard of recurrence during the follow-up was significantly (*p* < 0.05) lower with frovatriptan, as displayed in Fig. 1.

**Fig. 2:**
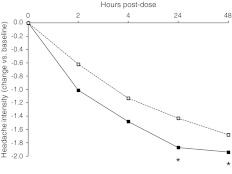
Changes in migraine intensity from baseline during treatment with frovatriptan (*continuous line*) or almotriptan (*dashed line*) in the 67 women with menstrually related migraine included in this analysis. The *asterisks* refer to the statistical significance of the between-treatment difference (*p < 0.05)

During the 48-h observation period, migraine intensity was progressively reduced by both drugs, with a statistically significant larger reduction (*p* < 0.05) with frovatriptan than with almotriptan at 24 and 48 h (Fig. 2).

## Discussion

In the present subgroup analysis of a double-blind, randomized, cross-over study [37], acute treatment of menstrually related migraine attacks with frovatriptan and almotriptan was associated with similar proportions of pain-relief and of pain-free episodes at 2, 4 and 24 h between the two drugs. However, frovatriptan showed significantly lower rates of headache recurrence over the 24 and 48 h than almotriptan. These results seem to indicate a more prolonged beneficial effect of frovatriptan compared to almotriptan, probably because of the different pharmacokinetics of the two drugs. Frovatriptan has a longer half-life than almotriptan (25–26 h vs. 3–4 h), which probably explains why frovatriptan has been reported to have a lower incidence of migraine recurrence than almotriptan [39–42]. Differences between the two drugs in this regard might be further enhanced in the subgroup of women with menstrually related migraine, who are known to suffer from a more disabling form of migraine as compared to non-menstrual migraineurs [1–3].

Results of our study corroborate those of previous randomized, placebo-controlled or open-label studies, which proved the efficacy of frovatriptan in the short-term prevention [28–32] or acute treatment of menstrual migraine [33–36]. Our results also provide more evidence and confirm results of previous almotriptan-based trials. In a retrospective analysis of a multicenter, multinational, randomized, double-blind parallel clinical trial, including 136 women treating a menstrual migraine attack with almotriptan, 68 % of subjects had obtained pain relief and 45 % were pain free at 2 h, while recurrence rates between 2 and 24 h after dosing were 33 % [13]. In another post hoc analysis of a multicenter, double-blind, parallel group, placebo-controlled trial almotriptan treatment in 97 women with menstrually related migraine resulted in 2 h pain relief, 2 h pain free and sustained pain-free rates of 77, 35 and 23 %, respectively [21]. Finally, in a recent randomized, double-blind, placebo-controlled cross-over study, including 74 premenopausal women with menstrually related migraine, almotriptan was associated with a significantly higher percentage of patients free of pain at 2 h compared with placebo (48 vs. 26 %). In this study, overall 36 % of patients were found to be sustained pain free with almotriptan versus 17 % with placebo [22]. The proportions achieved for study endpoints in almotriptan-treated patients in the trials cited above were slightly higher than those observed in our study.

Our study was the first directly comparing the efficacy of frovatriptan and almotriptan for the acute treatments for menstrually related migraine. In previous publications, frovatriptan showed pain-relief and pain-free rates at 2 and 24 h similar to those of rizatriptan and zolmitriptan, but significantly lower rates of recurrences at 24 h (15 vs. 22 % zolmitriptan and 10 vs. 32 % rizatriptan) [35, 36]. In another head-to-head double-blind, randomized trial comparing almotriptan with zolmitriptan, no significant differences between the two treatments were found, with similar pain-relief rates at 2 h (68 vs. 69 %), similar pain-free rates at 2 h (45 vs. 41 %) and similar recurrences at 2–24 h (33 vs. 35 %) [13].

In conclusion, results of the present analysis of data from a multicenter, randomized, double-blind, head-to-head, cross-over study suggest that frovatriptan and almotriptan are similarly effective in the immediate treatment of acute attack of menstrually related migraine. However, since frovatriptan seems to be superior to almotriptan in providing sustained relief from pain, this may suggest the use of the former in those patients with long-duration or high frequency of recurrence of migraine attacks. In the light of our results, we would indicate the use of frovatriptan in women with menstrual migraine, but with the caution of avoiding drug overuse or misuse, e.g. by limiting its use to more severe perimenstrual attacks. This will prevent occurrence of adverse events and progression and chronification of migraine, a risk which is directly related to the number of days with symptomatic drug intake.

We acknowledge that the retrospective nature of our analysis makes subsequent double-blind, randomized, prospective, large clinical trials mandatory to support our data.

## Appendix: List of study sites

Coordinator: Prof. B. Fierro (Palermo)

Investigators: M. Bartolini (Ancona), M.A. Giamberardino (Chieti), C. Lisotto (San Vito al Tagliamento), P. Martelletti (Roma), D. Moscato (Roma), B. Panascia (Catania) L. Savi (Torino), L.A. Pini (Reggio Emilia), G. Sances (Pavia), P. Santoro (Monza), G. Zanchin (Padova), B. Fierro (Palermo).
